# Evaluation of the inhibitory effect of various drugs / active ingredients on the activity of human diamine oxidase *in vitro*

**DOI:** 10.1186/2045-7022-4-S3-P23

**Published:** 2014-07-18

**Authors:** Roland Leitner, Eva Zoernpfenning, Albert Missbichler

**Affiliations:** 1 Scientific Association for Research and Education in the field of food-intolerances, Austria; 2 University of Applied Sciences, FH Campus Wien, Molecular Biotechnology Program, Austria; 3 SCIOTEC Diagnostic Technologies GmbH, R&D, Austria

## Background

In this study the influence of active ingredients of certain drugs on the activity of human diamine oxidase (DAO; EC 1.4.3.22) was quantified. DAO is the main enzyme in catabolism of biogenic amines in the intestine. Ingestion of food containing high amounts of biogenic amines in case of reduced activity of DAO leads to an accumulation of histamine which causes symptoms of histamine intolerance. Many drugs are suspected to inhibit DAO-activity, nevertheless, only few scientific data are available to support this thesis.

## Method

Therefore, based on a selection of drugs / active ingredients by literature research, the interaction with purified human diamine oxidase is determined and quantified in vitro with an activity test. Various drugs at pharmacologic concentrations were incubated with human diamine oxidase. Inhibition of diamine oxidase activity was calculated as the percentage of inhibition versus control (no inhibitor). To exclude drug formulation specific influences active ingredients (AI) of drug products (D) in pure form were examined.

## Results

Chloroquine and clavulanic acid showed greatest inhibition potential on diamine oxidase (> 90%). Cimetidine and verapamil showed inhibition of about 50%. Moderate influence on DAO was caused by isoniazid and metamizole, acetyl cysteine and amitriptyline (>20%). Diclofenac, metoclopramide, suxamethonium and thiamine have very low inhibition potential (<20%). Interestingly cyclophosphamide and ibuprofen displayed no effect on DAO.

## Conclusion

Since even levels of about 30% inhibition may be critical, most of the observed substances, can be designated as DAO inhibitors. Other drug components than active ingredients did not affect DAO activity or its interaction with a specific drug.

